# Bayesian Optimization for High-Dimensional Coarse-Grained Model Parameterization: A Case Study on Pebax Polymer

**DOI:** 10.1021/acs.jctc.5c01500

**Published:** 2026-01-21

**Authors:** Carlos A. Martins, Daniela A. Damasceno, Keat Yung Hue, Caetano Rodrigues Miranda, Erich A. Müller, Rodrigo A. Vargas-Hernández

**Affiliations:** † 28133 University of São Paulo, Department of Materials Physics and Mechanics, Institute of Physics, Rua do Matão 1371, São Paulo 05508-090, Brazil; ‡ University of São Paulo, Department of Mechatronics and Mechanical Systems Engineering, Polytechnic School, Av. Professor Mello Moraes, 2231, São Paulo 05315-970, Brazil; § 4615 Imperial College London, Department of Chemical Engineering, South Kensington Campus, London SW7 2AZ, U.K.; ∥ PETRONAS Research Sdn. Bhd., Lot 3288 & 3289, Off Jalan Ayer Itam, Kawasan Institusi Bangi, Kajang, Selangor 43000, Malaysia; ⊥ Department of Chemistry and Chemical Biology, 3710 McMaster University, 1280 Main Street West, Hamilton, Ontario L8S 4M1, Canada; # Brockhouse Institute for Materials Research, McMaster University, 1280 Main Street West, Hamilton, Ontario L8S 4M1, Canada

## Abstract

Coarse-grained (CG) force field models are extensively utilized in material simulations because of their scalability. Ordinarily, these models are parametrized using hybrid strategies that sequentially integrate top-down and bottom-up approaches. However, this combination restricts the capacity to jointly optimize all parameters. Although Bayesian optimization (BO) has been explored as an alternative search strategy to identify well-optimized CG parameters, its application has conventionally been limited to low-dimensional scenarios. This has contributed to the assumption that BO is unsuitable for more complex CG models, which often involve a large number of parameters. In this study, we challenge this assumption by successfully extending BO, using the tree-structured Parzen estimator (TPE) model, to optimize a high-dimensional CG model. Specifically, we show that a 41-parameter CG model of Pebax-1657, a copolymer composed of alternating polyamide and polyether segments, can be effectively parametrized using BO, resulting in a model that accurately reproduces the key physical properties of its parent atomistic representation. Our optimization framework simultaneously targets structural and thermodynamic properties, namely, density, radius of gyration, and glass transition temperature. Compared to traditional search algorithms, BO-TPE not only converges faster but also delivers consistent improvements over more standard parametrization approaches.

## Graphical abstract

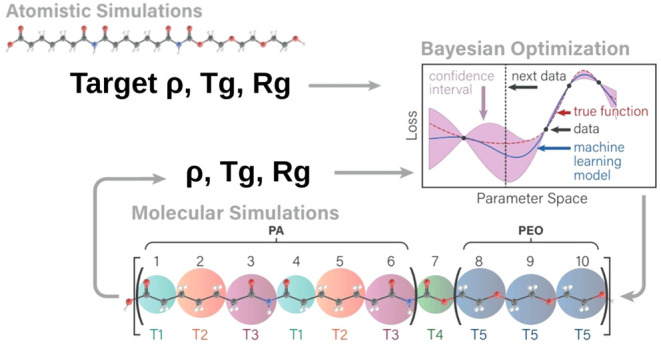

## 1 Introduction

Molecular dynamics (MD) is a well-known computational technique widely used to study and simulate physical and chemical phenomena.^1,2^ By employing molecular models such as all-atom or united-atom representations, MD has allowed prediction of the behavior of complex systems under a wide range of thermodynamic conditions.^3–5^ All-atom models offer the highest level of atomic detail by individually representing each atom, making them ideal for capturing phenomena governed by precise atomic-scale interactions.^6,7^ In contrast, united-atom models consolidate specific groups of atoms into a single interaction site, effectively simplifying the system and reducing computational demands. In both approaches, accuracy relies on the choice of force fields, which describe interatomic interactions and enable the calculation of structural, thermodynamic, mechanical, or transport properties. However, some phenomena extend beyond the typical size and time treated at the molecular scale, requiring a mesoscale perspective to capture their dynamics effectively. This necessity underscores the importance of coarse-grained (CG) models, as emphasized in several recent reviews.^8–10^

CG models simplify molecular representations by grouping atoms or functional groups into beads. This simplification inevitably results in a loss of chemical resolution, but when carefully designed, CG models can reproduce key properties of the system.^11^ Two main approaches guide the development of CG models: (i) bottom-up and (ii) top-down. Bottom-up frameworks derive CG parameters by mapping detailed quantum-level or atomistic information to a coarser scale, often using techniques such as iterative Boltzmann inversion,^12^ force matching^13^ or relative entropy.^14^ This family of methodologies emphasizes accuracy in reproducing molecular-level properties but lacks transferability across diverse thermodynamic conditions.^11^ In contrast, top-down approaches leverage experimental macroscopic data to optimize CG parameters directly, aiming for broader applicability. Advanced top-down methods include the MARTINI force field,^15^ widely used for biochemical applications, and the Statistical Associating Fluid Theory (SAFT-γ Mie) CG force field, which has found applications in fluid phase equilibria.^16,17^

Hybrid strategies combine both top-down and bottom-up methodologies in a sequential optimization process to develop CG models that are computationally efficient and capable of accurately representing molecular interactions. For example, Rahman et al.^6^ integrated the SAFT-γ Mie with atomistic simulations to model linear alkanes. The SAFT-γ Mie EoS represents molecules as chains of tangentially bonded spherical segments interacting via the Mie potential—a generalization of the Lennard-Jones potential that allows for independent tuning of repulsive and attractive forces. In the top-down optimization process, SAFT-γ Mie is employed to derive nonbonded interaction parameters by fitting experimental thermophysical data, such as densities, vapor pressures, and phase equilibria, ensuring that CG models align with macroscopic observations. The bottom-up stage complements this by refining bonded interactions through atomistic simulations, maintaining consistency with structural features at the molecular level.

As emphasized in previous works,^6^ the importance of accurately capturing both intra- and intermolecular interactions is critical for obtaining reliable predictions of thermodynamic properties such as vapor–liquid equilibrium and densities. Similarly, Fayaz-Torshizi and Müller^18^ applied these methodologies to polymers, demonstrating that incorporating detailed interactions significantly enhances the model’s ability to predict structural and dynamic behavior. By mapping atomistic configurations to CG beads, the models ensure that the bond lengths and angles closely reflect the equilibrium states observed experimentally. The harmonic potentials governing bond stretching and angular bending were calibrated using distributions of bond lengths and angles derived from atomistic simulations. These distributions were typically fitted with weighted Gaussian functions, ensuring consistency with key structural features such as end-to-end distances and radii of gyration. Although effective, this sequential optimization process may occur in isolated stages, providing no assurance of achieving a global solution, i.e., the optimal set of CG parameters. The decoupled nature of the optimization may lead to suboptimal CG models. It should be noted that, although decoupling the optimization problem in the development of CG models can lead to suboptimal parametrizations, there are successful examples of general-purpose CG force fields that employ this approach. A notable example is the SPICA family of force fields, based on the Shinoda-DeVane-Klein (SDK) multiproperty fitting framework,^19–21^ which provides one prominent example of such a hybrid strategy, demonstrating good transferability for surfactants, lipids, and biomolecular systems.

Machine learning (ML)-based CG models have also emerged as an alternative parametrization of CG models.^22–24^ However, despite significant progress, challenges remain, such as the need for extensive data sets and ensuring model transferability across diverse systems.^22^ Other alternative schemes based on ML models use Bayesian optimization (BO), a search algorithm, to identify the well-optimized parameters for CG models. BO is a powerful framework for design optimization, with successful applications spanning diverse fields such as robotics, environmental monitoring, and experimental design.^25^ In materials science, BO has proven highly effective across a range of tasks. For example, in the context of physical models, it has been employed to screen chemical compounds,^26–28^ minimize the energy of the Ising model,^29^ and optimize laser pulses for molecular control.^30^ In computational chemistry, BO has been applied to calibrate density functional models,^31^ refine physical models for the *cis*-*trans* photoisomerization of retinal in rhodopsin,^32,33^ and design potential energy surfaces for reactive molecular systems.^34^

BO provides a systematic and efficient approach for exploring high-dimensional parameter spaces,^35–42^ making it particularly well-suited for CG models of molecular and nanostructured materials, where traditional methods often struggle. Its application to CG models with low-dimensional parameter space has been explored in refs 43–45 , where the optimization process involved extracting physical properties from MD simulations and incorporating them into an objective function, typically defined as the sum of relative errors compared to an atomistic model or, by following a multiobjective optimization framework. The resulting CG models demonstrated a good agreement with their atomistic counterparts, highlighting BO’s potential for improving CG model development. However, these studies were restricted to CG models with relatively few parameters^43,44,46^ and did not fully account for all inter- and intramolecular interactions during optimization.

Due to recent studies demonstrating the successful application of BO in high-dimensional optimization problems involving up to several thousand parameters,^35–42^ we extend its use to the development of a system-specific CG model for Pebax. Unlike previous works limited to low-dimensional parameter spaces, our approach leverages BO’s scalability to address the complex, high-dimensional optimization landscape associated with this copolymer or similar systems. Here, we demonstrate that BO can efficiently optimize CG models with a substantially larger number of parameters, capturing both intra- and intermolecular interactions. In this system, the parameter search space exceeds 40 dimensions, underscoring BO’s scalability and its ability to navigate and refine intricate molecular interaction landscapes, ultimately enhancing the accuracy and versatility of CG models.

The remainder of this paper is organized as follows: Section 2 introduces the CG modeling framework for copolymers. Section 2.4 outlines the BO algorithm applied for CG model optimization, with emphasis on the physical properties used for calibration. Finally, Section 3 presents and discusses the results of our optimization strategy.

## 2 Coarse-Grained Models for Copolymers

### 2.1 Pebax Physical Model

Pebax copolymers,^47^ composed of alternating polyamide and polyether segments, are widely used in membrane technologies due to their tunable mechanical and transport properties. Pebax consists of polyamide (PA) and polyether (PE) segments, as shown in Figure 1. The PA segment, typically polyamide 6 (PA6) or polyamide 12 (PA12), provides mechanical strength and is covalently linked to the PE segment via ester groups.^48^ The PE component, which can be poly­(ethylene oxide) (PEO) or poly­(tetramethylene oxide) (PTMO),^48^ enhances gas transport and separation. As a case study, we focus on Pebax-1657, a specific grade composed of PEO and PA segments, as illustrated in Figure 2a. The repeat unit of the Pebax-1657 chain consists of 40% PA6 and 60% PEO,^49–51^ with each chain composed of one repeat unit only, as illustrated in Figure 2b. The PA-to-PEO ratio plays a crucial role in balancing mechanical flexibility and gas permeability, making Pebax-1657 particularly suitable for membrane applications such as CO_2_ capture and gas separation.^48,52^ Additionally, the incorporation of nanostructured materials, including MoS_2_ nanosheets,^53^ nonionic surfactants,^51^ zeolitic imidazolate frameworks-8 (ZIF-8) particles,^54^ and Azo@MOF-199–has been shown to enhance its adsorption and transport properties further.^52,56^ Given its technological potential and the challenges associated with fully atomistic simulations, Pebax-1657 is the polymer of choice in this work. An accurate coarse-grained (CG) model is essential for studying its structural and transport properties at larger scales.

**Figure 1:**
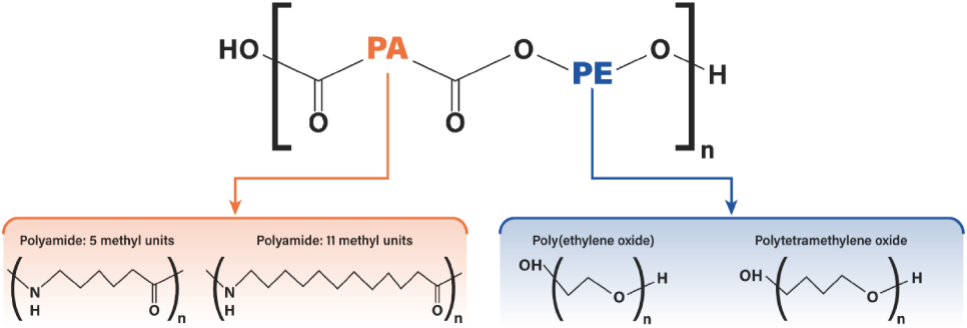
Chemical structure of Pebax and its polyamide (PA) and polyether (PE) segments.

**Figure 2:**
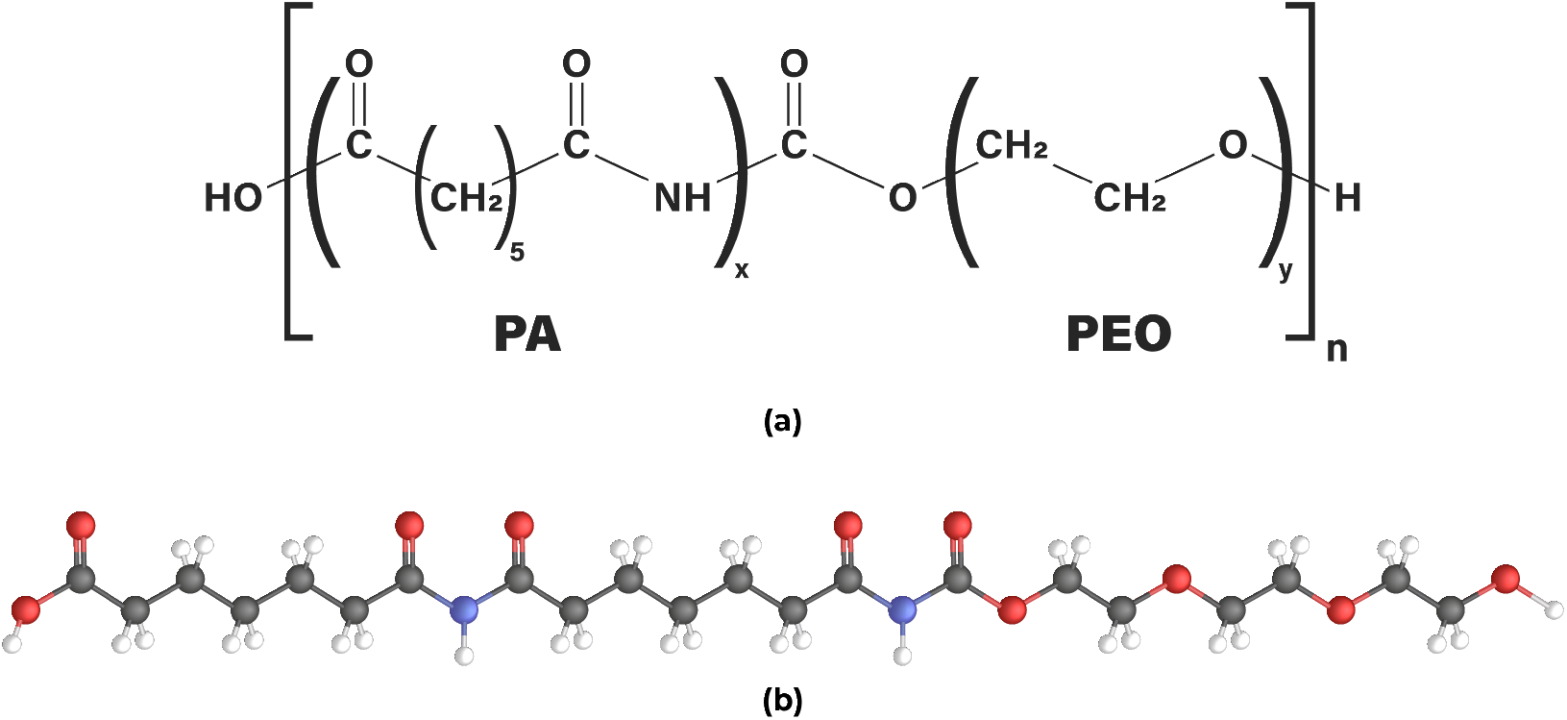
Panel (a), chemical structure of Pebax-1657 composed of PA6 and PEO segments. Panel (b), AA model of the Pebax-1657 chain with a composition of 40% PA6 and 60% PEO, corresponding to *n* = 1, x = 2, and y = 3. The gray spheres represent carbon atoms, while the red, blue, and white spheres represent oxygen, nitrogen, and hydrogen atoms, respectively.

The all-atom (AA) representation of the Pebax-1657 polymer was constructed using the Material Exploration and Design Analysis (MedeA) simulation software (version 3.5).^57^ The system consists of 50 polymer chains arranged in an amorphous configuration, generated using the Amorphous Builder module. The dimensions of the initial simulation box were set to 32.6 Å × 32.6 Å × 32.6 Å (length × width × height), yielding an experimental density of 1.136 g/cm^3^.^53^

### 2.2 Pebax CG Model

Given the technological relevance of Pebax-1657, an accurate coarse-grained (CG) model is essential for studying its structural and transport properties at larger scales. The CG model, shown in Figure 3, was developed based on available data from small oligomers, assuming the transferability of parameters. The atom-to-bead mapping followed a protocol to determine the intermolecular parameters of the CG model using the SAFT-γ Mie group contribution method. The model classifies the beads into five types: T1, T2, T3, T4, and T5, reflecting their chemical structure and functional roles within the polymer. The T1, T2, and T3 beads represent the polyamide segment, while T5 corresponds to the polyether segment. The T4 bead serves as a linker between the PA and the PEO regions.

**Figure 3:**
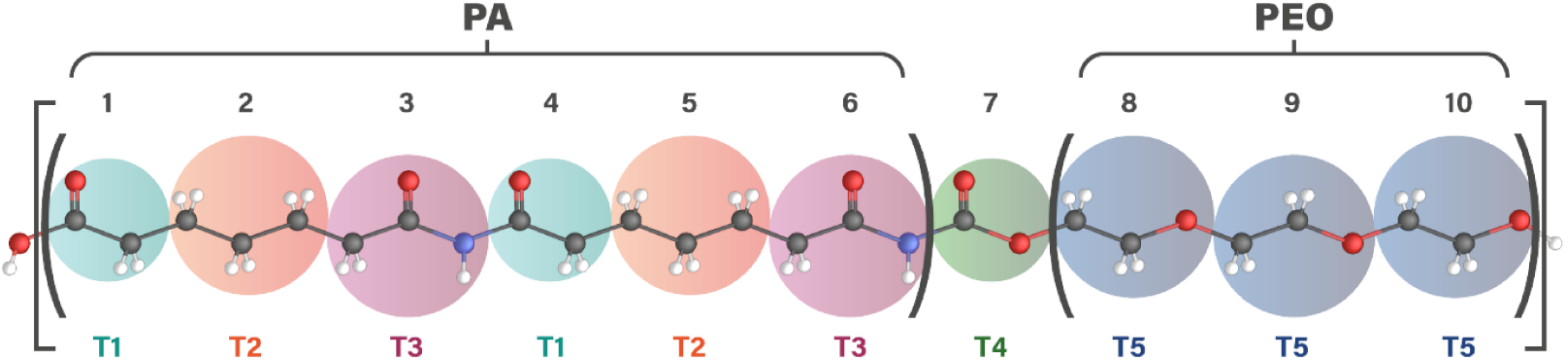
CG model of Pebax-1657 chain considering five distinct beads. The numbers 1 to 10 clearly visualize the structural organization and the total number of beads. The gray spheres represent carbon atoms, while the red, blue, and white spheres represent oxygen, nitrogen, and hydrogen atoms, respectively.

For this standard CG model, a hybrid strategy combining top-down and bottom-up methodologies was employed to derive both intermolecular and intramolecular FF parameters. Figure 4 summarizes the usual procedure to obtain the nonbonded intermolecular parameters [ϵ, σ, λ] using the SAFT-γ Mie group contribution method, where ϵ, σ, and λ represent the depth of the potential well, bead diameter, and Mie potential parameter that controls repulsion and attraction contributions, respectively. All intramolecular interactions were modeled using harmonic potentials for bond stretching and angle bending,^6^ and these parameters [*K*_*b*_, *K*_ϕ_, ϕ] were derived through fully atomistic simulations using MD simulations based on the PCFF+ potential.^58^

**Figure 4:**
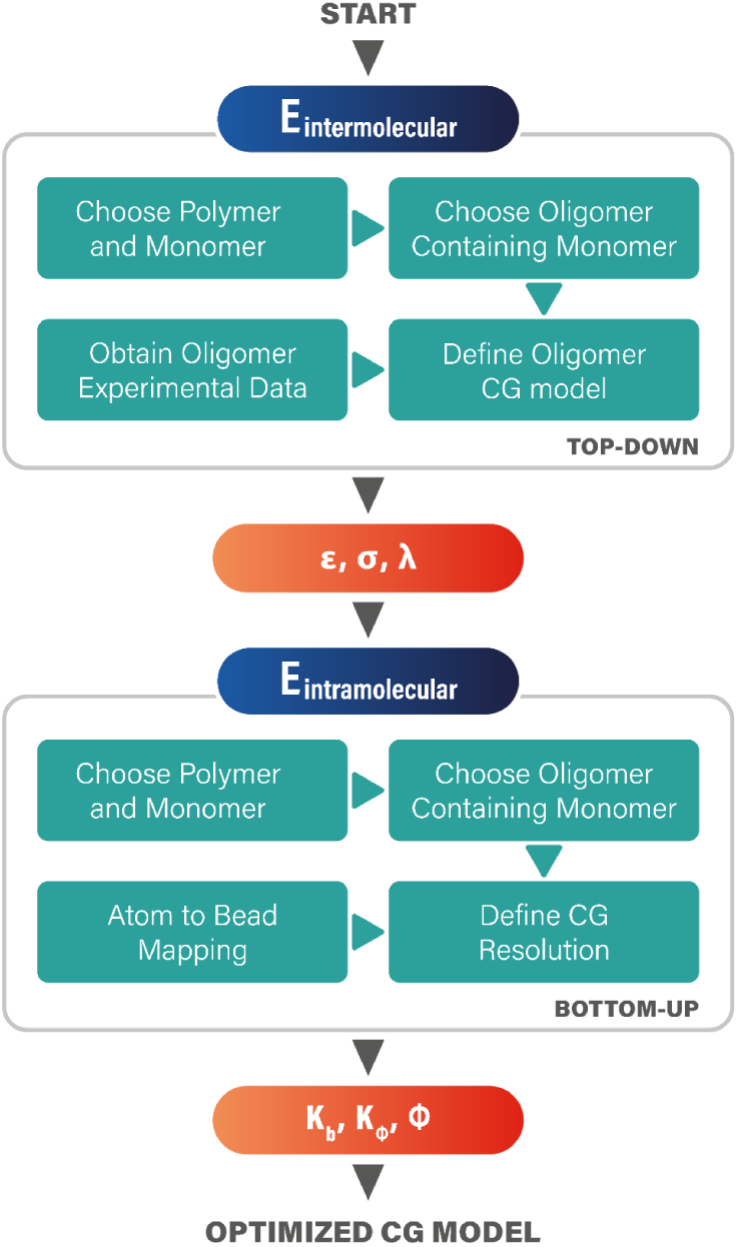
Hybrid strategy combining top-down and bottom-up methodologies to develop CG models.

In the SAFT formalism, the SAFT-γ Mie framework determines nonbonded intermolecular parameters by minimizing an objective function (L) that compares experimental data with predictions from the equation of state (EoS) for target properties such as saturated liquid densities and vapor pressures.^18^ This approach frames the determination of top-down interaction parameters as an optimization over macroscopic bulk properties.^16^ A Python-based implementation provides a practical framework for such optimizations.^59^

For simplicity, we employed Bottled SAFT.^60^ Bottled SAFT is a web application that provides SAFT-γ Mie parameters for a wide range of molecular fluids, allowing researchers to search for a specific molecule or upload a structure file to generate a set of parameters. Bottled SAFT is freely available at http://www.bottledsaft.org and is widely recognized for its accessibility and utility. For beads T1, T3, and T4, the parameters were obtained, considering heptanoic acid (CAS 111-14-8), *N*-methylacetamide (CAS 79-16-3), and pentyl pentanoate (CAS 2173-56-0), respectively. While Bottled-SAFT parameters derived from *n* segments are typically used to construct CG models for homonuclear molecules, several studies have adopted an alternative approach to build CG models. Notably, in the CG modeling of polystyrene,^61^ a single representative segment for toluene, from the *n* segments, was selected to construct the CG model. In line with this precedent, we believe that selecting a representative segment from each relevant molecule, along with its corresponding Bottled-SAFT parameters, offers a more accurate representation for our CG model. Meanwhile, data for bead T2 and the PEO segment (T5) were derived from previous studies of similar chain groups.^62^ For intramolecular force fields, all the steps illustrated in Figure 4 were performed to derive the bond stretching parameters (*K*_*b*_) and angle bending parameters ([*K*_ϕ_, ϕ]).

### 2.3 Bayesian Optimization

The development of CG models commonly depends on hybrid strategies that combine top-down and bottom-up methodologies, as shown in Figure 4. In these hybrid strategy approaches, the optimization is divided into two subproblems: (i) the intermolecular and (ii) the intramolecular parameters. First, the well-optimized parameters of the intermolecular potential [ϵ, σ, λ] are determined by minimizing the error between the reference data and the EoS calculations. Once the intermolecular parameters are optimized, a bottom-up approach follows to tune the intramolecular potential parameters, [*K*_*b*_, *K*_*ϕ*_, ϕ], for harmonic force fields.

Although hybrid approaches are successful,^6,16,60,63,64^ the division of the global optimization problem into separate subproblems can lead to inefficient exploration of the search space. This decomposition often results in nonoptimal solutions, as it fails to capture the interdependencies between parameter groups. To address this limitation, an alternative optimization approach is needed: one that explicitly accounts for these interdependencies while remaining computationally feasible, even for high-dimensional parameter spaces and complex physical systems. BO is often regarded as unsuitable for high-dimensional problems, primarily due to the challenge of constructing accurate surrogate models in large parameter spaces with limited data. This sense is reflected in prior applications to CG model optimization, where the number of parameters is typically limited to ∼10 or fewer.^43,44,46^ Despite these dimensional limitations, BO has been successfully applied to developing CG models by optimizing force fields based on physical properties extracted from molecular dynamics simulations.^43,44^ While existing applications of BO to CG models have been constrained to relatively low-dimensional problems, recent advances have demonstrated that BO can be effective in high-dimensional settings.^35–42^ For example, BO was successfully applied to a set of high-dimensional optimization problems involving up to 1,000 variables,^42^ demonstrating its scalability and adaptability to large, complex search spaces. These findings suggest that BO could be an effective tool for optimizing high-dimensional CG models and overcoming prior limitations of existing optimization algorithms, which do not fully account for all inter- and intramolecular interactions, thereby limiting the robustness of the resulting models.

For the Pebax polymer, we speculate that the specification of three key physical properties, namely (i) density (ρ), (ii) radius of gyration (*Rg*), and (iii) glass transition temperature (*Tg*) is sufficient to bootstrap the CG representation. The proposed optimization framework integrates these three properties into a single objective function,

1 $$L\left(\theta \right)=w_{\rho }L_{\rho }\left(\theta \right)+w_{Rg}L_{Rg}\left(\theta \right)+w_{Tg}L_{Tg}\left(\theta \right)$$

where **w** = [*w*_ρ_, *w*_*Rg*_, *w*_*Tg*_] are weight coefficients that balance the relative importance of each property. These weights prevent any single property from disproportionately influencing the optimization, which is particularly important here since density has more fitting points than the other properties. We used *w*_ρ_ = 1, *w*_*Rg*_ = 1,875, and *w*_*Tg*_ = 1.5 × 10^4^, see Supporting Information (SI) for more discussion regarding these values. The individual loss terms in eq 1 are computed as relative errors between the CG model predictions and the reference data,

2 $$L_{\rho }\left(\theta \right)=\overset{N_{\rho }}{\underset{i=1}{\sum }}{\left(\frac{\rho \left(T_{i},\theta \right)-\mathrm{\rho ̂}\left(T_{i}\right)}{\mathrm{\rho ̂}\left(T_{i}\right)}\right)}^{2}$$

3 $$L_{Rg}\left(\theta \right)=\overset{N_{Rg}}{\underset{i=1}{\sum }}{\left(\frac{Rg\left(T_{i},\theta \right)-\mathrm{R̂}g\left(T_{i}\right)}{\mathrm{R̂}g\left(T_{i}\right)}\right)}^{2}$$

4 $$L_{Tg}\left(\theta \right)={\left(\frac{Tg\left(\theta \right)-\mathrm{T̂}g}{\mathrm{T̂}g}\right)}^{2}$$

where *T*_*i*_ denotes the temperatures at which MD simulations were performed, while *N*_ρ_ and *N*_*Rg*_ represent the number of data points used for density and radius of gyration calculations, respectively. These properties play distinct roles in the validation of CG models. The density provides direct experimental comparability and is directly obtained in MD. The radius of gyration reflects the spatial conformation and compactness of polymer chains, and the glass transition temperature integrates thermodynamic and structural information, offering insight into thermal behavior.

For the copolymer Pebax, $L_{\rho }$ is influenced by the glass temperature. To ensure representative sampling, we selected eight discrete temperatures $\left(\{T_{i}{\}}_{i=1}^{8}\right)$ , four below and four above the glass transition temperature observed in the atomistic model; see Figure S1 in the SI. The glass transition temperature was then determined as the intersection of these two fitted curves, following the methodology outlined by Patrone et al.^65^ Further numerical details are provided in the SI. Temperatures near the glass transition were excluded, as the density–temperature relationship is discontinuous at the glass temperature.^65^ We used density measurements at each of the eight temperatures to fit two linear curves. This approach provided a denser set of target points on either side of the glass transition, refining the density–temperature relationship for optimization. Initially, we included points near the glass transition in the fits. However, we found that excluding them led to improved results. The glass transition temperature was then determined as the intersection of these two fitted curves, following the methodology outlined by Patrone et al.^65^

For $L_{Rg}$ , the radius of gyration component, we also ensured reliable sampling by simulating a single Pebax chain instead of a fully populated polymer box. This approach mitigated the risk of the sampling of the radius of gyration becoming confined to a limited region of phase space, which could otherwise lead to inaccurate property estimates. By isolating a single chain, we enhanced sampling across phase space. The radius of gyration was then computed at 15 different temperature points. Additional details on the MD simulations are provided in the SI. All target properties used as reference data in eq 1 were determined using an atomistic model based on the PCFF+ force field. The data supporting the findings of this study are openly available in the following https://github.com/camjjr/bo_cgff. The parameters of the atomistic model used in this work can be found in the following link.

### 2.4 Tree-Structured Parzen Estimator

Grounded in Bayesian inference, BO systematically balances exploration and exploitation by iteratively updating a surrogate model that navigates the search space efficiently. By leveraging information from previous evaluations, BO aims to identify the most favorable regions and direct the search toward the global optimum. Two key components constitute the core of BO: the surrogate model, which approximates the objective function, and the acquisition function, which guides the search. We used as a surrogate model the Tree-structured Parzen Estimator (TPE).^66^ Unlike traditional Gaussian process-based approaches, TPE constructs a nonparametric density estimator that partitions the search space into two regions: (i) a top quantile of observations with favorable objective function values and (ii) a lower quantile containing the remaining observations.^66^ This structure allows TPE to model the search space adaptively, prioritizing promising regions while maintaining diversity in exploration. Formally, TPE models^66^ parametrize the conditional probability distribution as,

5 $$p\left(\theta |y,D\right)=\left\{ \begin{array}{ll} p\left(\theta |D^{w}\right) & y\leq y^{\gamma }\\ p\left(\theta |D^{b}\right) & y>y^{\gamma } \end{array}\right.$$

where *p*(**θ**|*y*, *D*) represents the probability density function of the parameters, and *D* is the set of observed objective function values {*y*_1_, *y*_2_, *y*_3_, ···, *y*_*n*_} obtained from evaluations of eq 1. The subsets *D* ^*b*^ and *D* ^*w*^ correspond to the best (b) observations and worst (w), respectively. These groups are dynamically updated at each iteration based on the hyperparameter γ, which controls the balance between exploration and exploitation.

The probability density function within each group is estimated as follows,

6 $$p\left(\theta |D^{i}\right)=\omega_{0}^{i}p_{0}\left(\theta \right)+\overset{N_{i}}{\underset{n=1}{\sum }}\omega_{n}^{i}k_{n}\left(\theta ,\theta_{n}|b^{i}\right)$$

where ω^*i*^ represents the weights assigned to observations, *i* denotes either the best (b) or worst (w) group, and *N*_*i*_ is the number of observations within the group. The term *p*_0_ defines a prior distribution that influences the level of exploration, while *k*_*n*_(**θ**, **θ**_**n**_|*b* ^*i*^) is the kernel function used for density estimation, employing a Gaussian kernel for numerical variables and an Aitchison-Aitken kernel for categorical variables. *b* ^*i*^ is simply the bandwidth for each group. For this study, we used the following acquisition function,

7 $$P\left(y\leq y^{*}|\theta ,\;D\right)=\int_{-\infty }^{y^{*}}p\left(y|\theta ,\;D\right)dy$$

Using eq 5, the acquisition function is,^66^

8 $$P\left(y\leq y^{*}|\theta ,D\right)\overset{\mathrm{rank}}{≃}r\left(\theta |D\right)=\frac{p\left(\theta |y,D^{b}\right)}{p\left(\theta |y,D^{w}\right)}$$

which is equivalent to the well-established PI acquisition function.^66,67^ The TPE model and the BO algorithm were both implemented using the Optuna library.^68^

## 3 Results

### 3.1 CG Optimization

Optimization of CG models, even with BO, is a dynamic process, as at each iteration BO infers more about L(θ) . Figure 5a illustrates the lowest value of L found by BO as a function of the iterations. To demonstrate BO’s robustness, we considered three independent runs, each with an initial random **θ**. All runs successfully converged to similar optimal solutions with similar L , in less than 600 iterations (≈2000 min), as shown in Figure 5a. Furthermore, the optimal solutions identified in each run exhibit objective function values several orders of magnitude lower than the initial guesses, and by the hybrid strategy. These results suggest that BO effectively avoids regions where L is high (red points in Figure 5b) while concentrating trials in the low areas (blue points in Figure 5b). This is further supported by the histogram of the sampled values of L of a single BO run; insight panel in Figure 5a.

**Figure 5:**
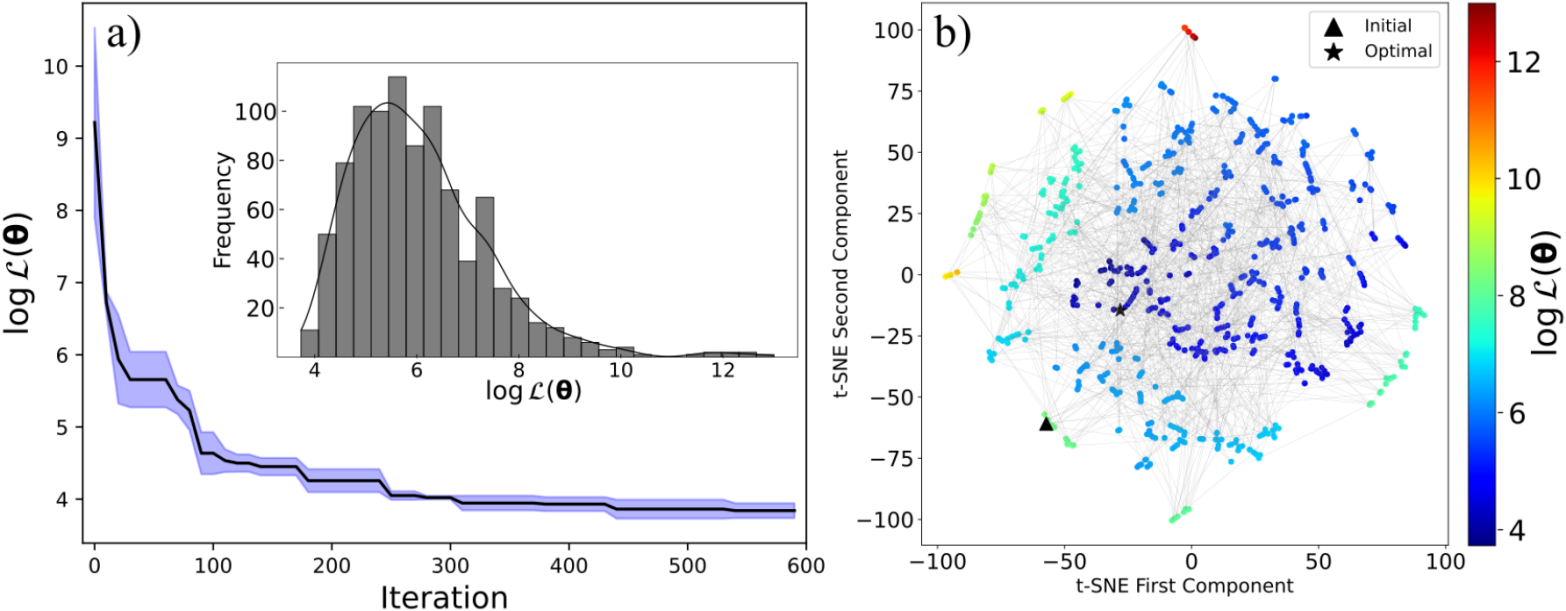
(Panel a) Convergence of BO as a function of the iterations. The solid blue line and shaded blue region indicate the logarithm of L(θ) (eq 1) and the standard deviation computed with three independent optimization trajectories. The insight is the histogram of the sampled values of log⁡⁡L(θ) of one of the BO trajectories. (Panel b) t-SNE visualization of the sampled parameters of a single BO run. Each point represents a sampled parameter set, color-coded by its total objective function value. ▲ and ★ represent the parameters of the initial and best-found CG model. We used a logarithmic scale to improve the visualization of the ranges for each property’s error function.

It is important to emphasize the capability of this CG model, which consists of 41 parameters. Conventional search methods, such as grid-based approaches, typically require more than 10 trials or grid points per parameter of the CG model. Given the complexity of the Pebax CG model, a grid search approach would need more than 10^40^ iterations, an infeasible number considering the computational cost of MD simulations to evaluate the three target physical properties. Recent studies have also demonstrated the efficiency of BO in optimizing CG models.^43,44^ These previous studies focused on simpler CG models, resulting in lower-dimensional optimization problems. To our knowledge, this study is the first to optimize all parameters of a complex CG model, resulting in a high-dimensional optimization problem, due to the number of parameters in the CG model. The results demonstrate that BO-TPE remains effective even for optimization problems with more than a dozen parameters, reinforcing its suitability for complex CG model development.

To understand why BO is particularly effective for the Pebax CG model, we investigated whether its 41 parameters could be represented in a lower-dimensional space. We first applied Principal Component Analysis (PCA) to the parameters sampled by BO (Figure 6), finding that 90% of the variance is captured by the first 28 principal components. While this only reduces the dimensionality by about 32%, it does indicate some redundancy. To probe for deeper structure, we also performed an ISOMAP analysis, but it did not uncover clear patterns linking low-loss regions, suggesting the absence of a simple nonlinear manifold. Repeating the PCA on samples with log⁡⁡L<5.5 slightly improved compression: 23 components sufficed to explain 90% of the variance (Figure 6a). This modest gain suggests that low-loss regions exhibit somewhat more structure. Recent work supports the idea that BO can benefit from linear embeddings in high-dimensional spaces.^69^ Our results align partially with this view—parameter correlations do emerge in regions of low objective, but retaining 65% of the original dimensions remains necessary. Overall, these findings suggest that BO’s success here stems less from a highly compressible geometry and more from its ability to adaptively explore a moderately structured, high-dimensional space with some redundancy but no sharply defined low-dimensional manifold.

**Figure 6:**
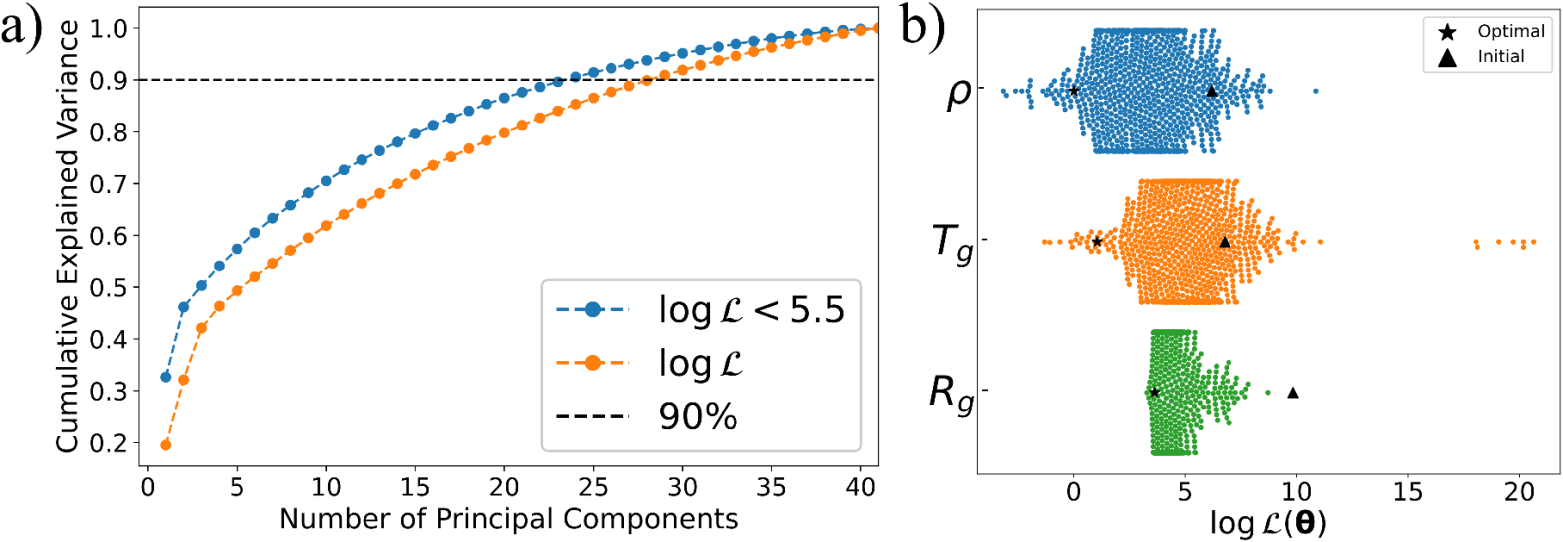
Panel (a) Cumulative explained variance as a function of the number of principal components for the CG parameters sampled by BO. The variance curves indicate that most of the variability in the optimized parameter space can be captured by a reduced number of effective dimensions; 20 to 25 components account for 90% of the variance. Panel (b) Distribution of sampled parameters obtained by BO for each component of the total objective function (eq 1). The ▲ and ★ symbols denote the initial parameter set and the optimal configuration found by BO, respectively.

While the objective function in eq 1 aggregates multiple target properties into a single global function, the CG model’s optimization can alternatively be approached as a multiobjective optimization problem. In this case, the goal is to identify the Pareto front, which characterizes the trade-offs between competing objectives.^70–72^ Although this approach provides a richer understanding of the parameter space, it is less practical for high-dimensional CG models, as this optimization framework requires a greater number of evaluations. Given the linear combination nature of the total objective function, we also analyze the sampled values of each component of L . Figure 6b presents the individual values of $L_{\rho }$ , $L_{Tg}$ , and $L_{Rg}$ sampled by BO. These results show that BO effectively samples low values $\left(\log L_{i}<5\right)$ for all three components, without a clear preference for any one. Furthermore, these results also indicate that density and glass transition temperature exhibit lower overall relative errors compared to the radius of gyration, suggesting that *Rg* is the most challenging physical property to reproduce. The relatively low errors observed for density and glass transition temperature in some of the different sampled parameters of the CG model (each point in Figure 6b) may be attributed to their intrinsic relationship, as *Tg* can be determined from the slopes of the density curve; for more details, see refs 65,73 . Overall, our findings suggest that none of the physical properties overwhelmingly dominate the objective function and indicate that BO successfully balances its optimization. This balance enhances the robustness of the resulting coarse-grained models, ensuring a more reliable reproduction of the target physical properties.

### 3.2 Physical Properties

Figure 7 compares the density and radius of gyration for a single Pebax chain, as predicted by three different models: the atomistic reference model, the CG-BO model, and the CG-Hybrid Strategy model, the latter was developed using a hybrid optimization approach described in the SI. The well-optimized parameter set **θ** obtained by BO is reported in the SI, referred to as the CG-BO model. To mitigate the effects of discontinuity in the density profile, only four temperature below the glass temperature (150 K < *T* < 225 K) and four high-temperature above it (425 K < *T* < 500 K) were considered in the optimization, as detailed in SI.

**Figure 7:**
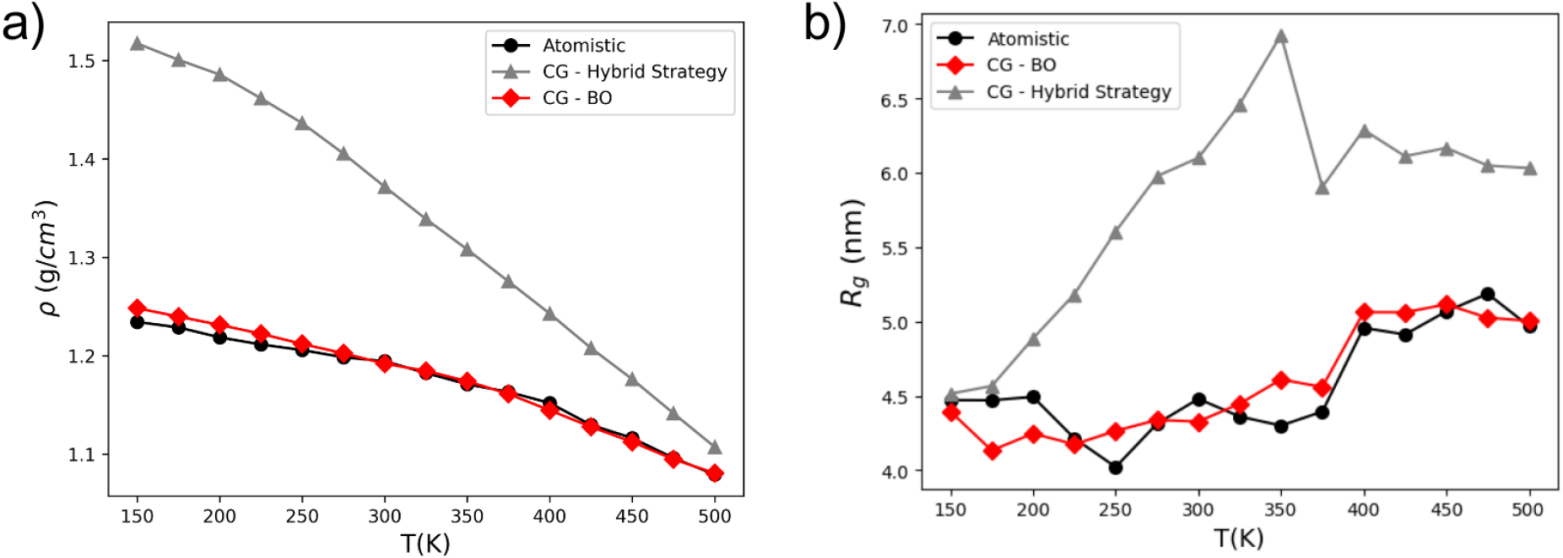
Comparison of the density (Panel a) and the radius of gyration (Panel b) for a single Pebax chain using the atomistic model as a reference, alongside the CG-hybrid strategy and CG-BO models.

Regarding the density (Figure 7a), the CG-BO model closely follows the atomistic reference, with only minor deviations at low temperatures (*T* < 250 K). In contrast, the CG-Hybrid Strategy model exhibits significant discrepancies across the entire temperature range, suggesting poor transferability of its parameters, which were originally derived from different polymer systems. It is worth mentioning that the density of the atomistic model deviates from the experimental measurements by 4.3%.^53^ The predicted radius of gyration (Figure 7b) further highlights the limitations of the hybrid approach. The CG-Hybrid Strategy model significantly deviates from the atomistic reference, particularly at temperatures above 200 K. Conversely, the CG-BO model shows strong agreement with the reference data, effectively capturing the general trend of *Rg*. This demonstrates BO’s capability to systematically explore the parameter space and identify a coarse-grained model that retains key structural characteristics, even within a high-dimensional optimization setting.

For the glass transition temperature (*Tg*), Table 1 summarizes the predicted values obtained from the three models. Using the atomistic model as a reference, once again, the CG-BO approach outperforms the CG-Hybrid strategy, producing a significantly lower relative error (10.95%), while the CG-Hybrid strategy model exhibits a relative error of 34.62%.

**Table 1:** Predicted Glass Transition Temperature and Its Relative Error $\left(L_{tg}\left(\theta \right)\right)$ with the CG-BO and CG-Hybrid Strategy Models^a^

Model	*Tg* (K)	$L_{Tg}\left(\theta \right)$
Atomistic	378.10	-
CG-BO	336.69	0.012
CG-Hybrid Strategy	247.18	0.119

a We use an atomistic model as the reference.

Lastly, we investigated the sensitivity of the objective function (eq 1) to changes in the weight parameters **w** = [*w*_ρ_, *w*_*Rg*_, *w*_*Tg*_], given our choice of a single-objective optimization approach over a full multiobjective framework. Although multiobjective optimization, as proposed by Sestito et al.^44^ is a viable alternative for CG model tuning; it would require significantly more evaluations of L and MD simulations, which becomes impractical for high-dimensional CG models. Furthermore, such an approach would yield a set of Pareto-optimal solutions, making model selection less straightforward if no clearly dominant Pareto point exists.

Figure 8 shows the objective space corresponding to two different sets of weight combinations, **w**_2_ = [10, 1875, 1.5 × 10^3^] and **w**_3_ = [5, 18750, 1.5 × 10^3^], where we deliberately shifted the balance between the three physical properties. Each resulting CG model is denoted CG-BO­(**w**_*i*_). As seen in Figure 8a and b, all three weight configurations lead to similarly accurate predictions for ρ and *Rg*. Figure 8c and d show that each **w**_*i*_ emphasizes a different region of the individual loss components, yet the joint performance remains consistent. The predicted glass transition temperature in all three optimization cases deviates by 82.94 K for **w**_2_ and 37.01 for **w**_3_ from the atomistic model. Overall, the CG-BO­(**w**_3_) reproduces more accurately ρ and *Tg*, but predicted *Rg* is ∼30% less accurate than CG-BO­(**w**). These results indicate that the optimization landscape is relatively robust to moderate changes in **w** and that the BO-CG model is flexible enough to capture relevant features regardless of the precise weighting scheme. For the **W**_2_ and **W**_3_ models, BO-TPE also converged to the well-optimized parameters in fewer than 600 total iterations; see Figure S2 in the SI.

**Figure 8:**
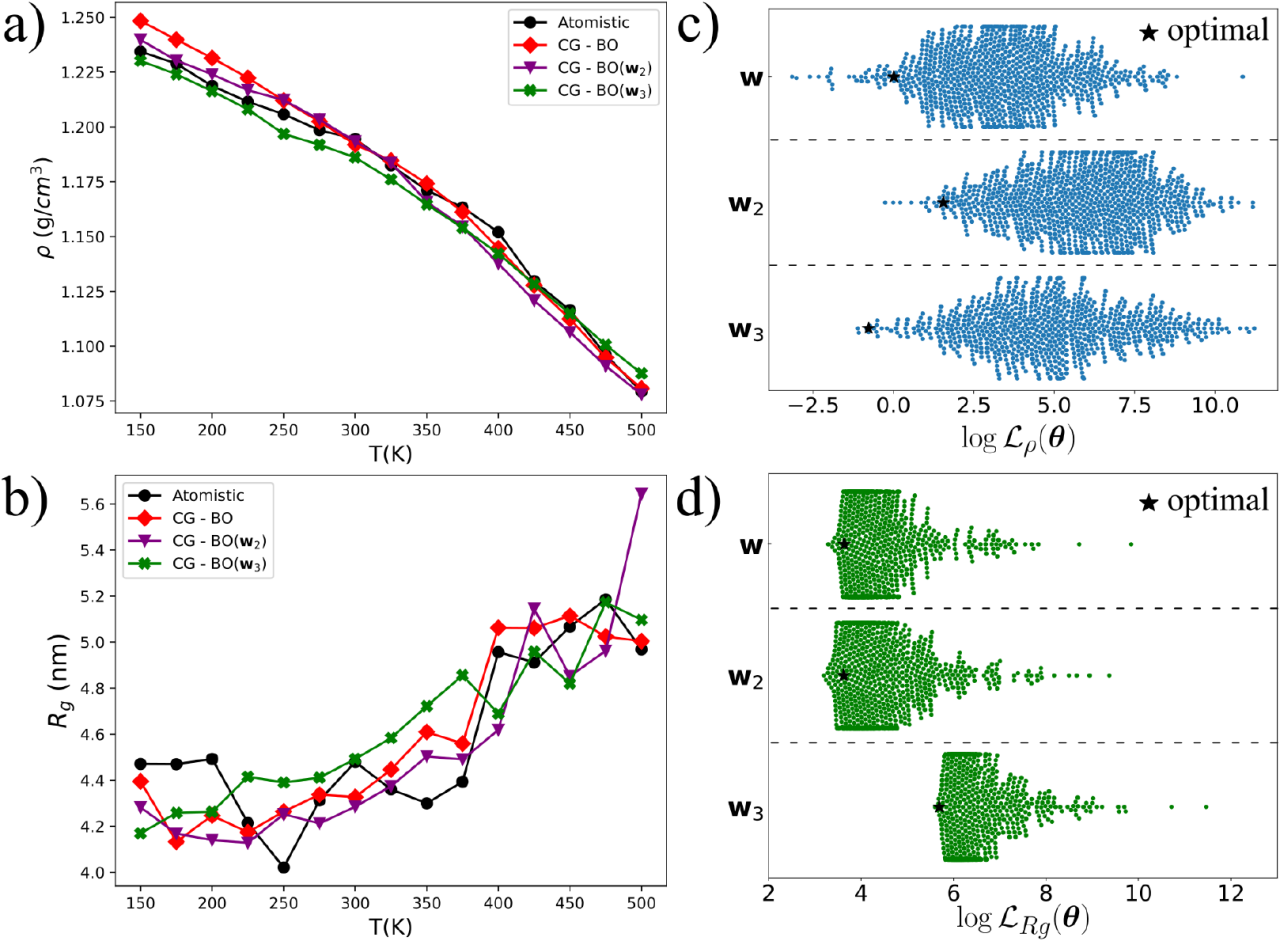
Panels (a) and (b) show the predicted density and radius of gyration, respectively, for the three optimal CG models, CG-BO­(**w**_*i*_), obtained using different weight vectors **w**_*i*_ in the total loss function. Panels (c) and (d) display the sampled values of $\log L_{\rho }$ and $\log L_{R_{g}}$ corresponding to each optimization. ★ symbols mark the final CG-BO model obtained for each choice of **w**. Each optimization ran for 600 iterations to ensure convergence. See the main text for further details.

Despite the demonstrated scalability and efficiency of the proposed BO-TPE framework, two main limitations remain. First, as with most Bayesian optimization methods, the accuracy and generality of the approach depend on the design of the objective function itself, as it defines how model performance is quantified across the parameter space. Second, the intrinsic cost of evaluating the objective function, which in our case involves molecular simulations. The current implementation was tailored to polymer systems through their glass-transition behavior and would require adaptation to other material classes or force fields. Future work will focus on developing transferable objective functions that extend the applicability of this framework to broader families of CG models.

## 4 Conclusion

We demonstrated that Bayesian optimization enables the efficient optimization of coarse-grained models with a large number of parameters. Unlike conventional optimization methods that often rely on parameter-splitting heuristics to navigate complex search spaces, BO adopts a fundamentally different strategy, using acquisition functions to guide efficient sampling and globally explore the parameter space. Using BO, we optimized a CG model for the copolymer Pebax without decomposing the problem into smaller subtasks, as is typically done in CG model development. Remarkably, BO-TPE required fewer than 600 iterations to converge to an optimal set of 41 parameters. The resulting CG model accurately reproduces key physical properties, showing strong agreement with atomistic simulations. It is important to emphasize that, although we used atomistic simulations as the reference in this study, the proposed methodology can be readily adapted to optimize CG models against experimental data.

This work is motivated by recent advances in extending BO to high-dimensional settings, highlighting its potential beyond low-dimensional parameter tuning. As BO techniques continue to evolve, particularly with the development of trust-region strategies and multifidelity frameworks, we anticipate that these tools will further improve the scalability and efficiency of CG model optimization, paving the way for faster, data-driven materials discovery and molecular design.

## Supplementary Material



## Notes

Numerical details of the molecular dynamics simulations; definition of the objective function and weighting scheme used in Bayesian optimization; optimal coarse-grained force-field parameters obtained from Bayesian optimization and hybrid strategies; convergence analysis under different weight configurations; predicted glass transition temperatures compared with atomistic references; principal component analysis (PCA) and t-SNE analysis of optimized parameter sets (PDF)

## References

[1] Hollingsworth S. A., Dror R. O. (2018). Molecular dynamics simulation for all. Neuron.

[2] Karplus M., McCammon J. A. (2002). Molecular dynamics simulations of
biomolecules. Nat. Struct. Biol..

[3] Sadeghi M., Noé F. (2020). Large-scale
simulation of biomembranes incorporating
realistic kinetics into coarse-grained models. Nat. Commun..

[4] Kostritskii A. Y., Machtens J.-P. (2021). Molecular mechanisms
of ion conduction and ion selectivity
in TMEM16 lipid scramblases. Nat. Commun..

[5] Ding X., Lin X., Zhang B. (2021). Stability
and folding pathways of tetra-nucleosome
from six-dimensional free energy surface. Nat.
Commun..

[6] Rahman S., Lobanova O., Jiménez-Serratos G., Braga C., Raptis V., Müller E. A., Jackson G., Avendano C., Galindo A. (2018). SAFT-*γ* Force Field for the simulation
of molecular fluids. 5. Hetero-group coarse-grained models of linear
alkanes and the importance of intramolecular interactions. J. Phys. Chem. B.

[7] Avendaño C., Lafitte T., Galindo A., Adjiman C. S., Jackson G., Müller E. A. (2011). SAFT-*γ* force
field for the simulation
of molecular fluids. 1. A single-site coarse grained model of carbon
dioxide. J. Phys. Chem. B.

[8] Joshi S. Y., Deshmukh S. A. (2021). A review of advancements
in coarse-grained molecular
dynamics simulations. Mol. Simul..

[9] Noid W. G. (2023). Perspective:
Advances, challenges, and insight for predictive coarse-grained models. J. Phys. Chem. B.

[10] Brini E., Algaer E. A., Ganguly P., Li C., Rodríguez-Ropero F., van der Vegt N. F. (2013). Systematic
coarse-graining methods for soft matter
simulations–a review. Soft Matter.

[11] Potter T. D., Tasche J., Wilson M. R. (2019). Assessing
the transferability of
common top-down and bottom-up coarse-grained molecular models for
molecular mixtures. Phys. Chem. Chem. Phys..

[12] Reith D., Pütz M., Müller-Plathe F. (2003). Deriving effective
mesoscale potentials from atomistic simulations. J. Comput. Chem..

[13] Izvekov S., Parrinello M., Burnham C. J., Voth G. A. (2004). Effective force
fields for condensed phase systems from ab initio molecular dynamics
simulation: A new method for force-matching. J. Chem. Phys..

[14] Carmichael S. P., Shell M. S. (2012). A New Multiscale Algorithm and Its Application to Coarse-Grained
Peptide Models for Self-Assembly. J. Phys. Chem.
B.

[15] Souza P. C., Alessandri R., Barnoud J., Thallmair S., Faustino I., Grünewald F., Patmanidis I., Abdizadeh H., Bruininks B. M., Wassenaar T. A. (2021). Martini 3: A general purpose force field for
coarse-grained molecular
dynamics. Nat. Methods.

[16] Müller E. A., Jackson G. (2014). Force-field parameters
from the SAFT-*γ* equation of state for use in
coarse-grained molecular simulations. Annu.
Rev. Chem. Biomol. Eng..

[17] Herdes C., Totton T. S., Müller E. A. (2015). Coarse
grained force field for the
molecular simulation of natural gases and condensates. Fluid Phase Equilib..

[18] Fayaz-Torshizi M., Müller E. A. (2022). Coarse-Grained Molecular Simulation
of Polymers Supported
by the Use of the SAFT-*γ*$\gamma$ Mie Equation
of State. Macromol. Theory Simul..

[19] Kawamoto S., Liu H., Miyazaki Y., Seo S., Dixit M., DeVane R., MacDermaid C., Fiorin G., Klein M. L., Shinoda W. (2022). SPICA Force
Field for Proteins and Peptides. J. Chem. Theory
Comput..

[20] Shinoda W., DeVane R., Klein M. L. (2007). Multi-property fitting and parameterization
of a coarse grained model for aqueous surfactants. Mol. Simul..

[21] Singh A. P., Tanaka H., Miyazaki Y., Shinoda W. (2025). SPICA Force
Field for
Nucleic Acids and Its Application to Lipid Nanoparticles. J. Phys. Chem. B.

[22] Durumeric A. E., Charron N. E., Templeton C., Musil F., Bonneau K., Pasos-Trejo A. S., Chen Y., Kelkar A., Noé F., Clementi C. (2023). Machine learned coarse-grained protein force-fields:
Are we there yet?. Curr. Opin. Struct. Biol..

[23] Unke O. T., Chmiela S., Sauceda H. E., Gastegger M., Poltavsky I., Schütt K. T., Tkatchenko A., Müller K.-R. (2021). Machine Learning Force Fields. Chem. Rev..

[24] Röcken S., Zavadlav J. (2024). Accurate machine learning force fields
via experimental
and simulation data fusion. npj Comput. Mater..

[25] Shahriari B., Swersky K., Wang Z., Adams R. P., de Freitas N. (2016). Taking the
Human Out of the Loop: A Review of Bayesian Optimization. Proc. IEEE.

[26] Ueno T., Rhone T. D., Hou Z., Mizoguchi T., Tsuda K. (2016). COMBO: An efficient Bayesian optimization library for materials science. Mater. Discovery.

[27] Jalem R., Kanamori K., Takeuchi I., Nakayama M., Yamasaki H., Saito T. (2018). Bayesian-driven first-principles
calculations for accelerating exploration
of fast ion conductors for rechargeable battery application. Sci. Rep..

[28] Ju S., Shiga T., Feng L., Hou Z., Tsuda K., Shiomi J. (2017). Designing nanostructures for phonon
transport via Bayesian
optimization. Phys. Rev. X.

[29] Tamura R., Hukushima K. (2018). Bayesian optimization for computationally
extensive
probability distributions. PLoS One.

[30] Deng Z., Tutunnikov I., Averbukh I. S., Thachuk M., Krems R. V. (2020). Bayesian
optimization for inverse problems in time-dependent quantum dynamics. J. Chem. Phys..

[31] Vargas-Hernández R. A. (2020). Bayesian
Optimization for Calibrating and Selecting Hybrid-Density Functional
Models. J. Phys. Chem. A.

[32] Vargas-Hernández R. A., Chuang C., Brumer P. (2021). Multi-objective optimization for
retinal photoisomerization models with respect to experimental observables. J. Chem. Phys..

[33] Singh D., Chuang C., Brumer P. (2024). Machine Learning
Optimization of
Non-Kasha Behavior and of Transient Dynamics in Model Retinal Isomerization. J. Phys. Chem. Lett..

[34] Vargas-Hernández R. A., Guan Y., Zhang D. H., Krems R. V. (2019). Bayesian optimization
for the inverse scattering problem in quantum reaction dynamics. New J. Phys..

[35] Kandasamy, K. ; Schneider, J. ; Poczos, B. High Dimensional Bayesian Optimisation and Bandits via Additive Models. In Proceedings of the 32nd International Conference on Machine Learning; PMLR: Lille, France, 2015, pp. 295–304.

[36] Wang Z., Hutter F., Zoghi M., Matheson D., De Freitas N. (2016). Bayesian optimization
in a billion dimensions via random embeddings. J. Artif. Intell. Res..

[37] Nayebi, A. ; Munteanu, A. ; Poloczek, M. A Framework for Bayesian Optimization in Embedded Subspaces. In Proceedings Of The 36th International Conference On Machine Learning; PMLR, 2019, pp. 4752–4761.

[38] Eriksson, D. ; Pearce, M. ; Gardner, J. ; Turner, R. D. ; Poloczek, M. Scalable Global Optimization via Local Bayesian Optimization. In Advances In Neural Information Processing Systems; NeurIPS Proceedings, 2019; Vol.: 32.

[39] Eriksson, D. ; Jankowiak, M. High-dimensional Bayesian optimization with sparse axis-aligned subspaces. In Proceedings Of The Thirty-Seventh Conference On Uncertainty In Artificial Intelligence; PMLR, 2021, pp. 493–503.

[40] Papenmeier, L. ; Nardi, L. ; Poloczek, M. Increasing the Scope as You Learn: Adaptive Bayesian Optimization in Nested Subspaces Advances In Neural Information Processing Systems NeurIPS Proceedings 2022 35 11586–11601

[41] Ziomek, J. K. ; Ammar, H. B. Are Random Decompositions all we need in High Dimensional Bayesian Optimisation? In Proceedings Of The 40th International Conference On Machine Learning; PMLR, 2023, pp. 43347–43368.

[42] Hvarfner, C. ; Hellsten, E. O. ; Nardi, L. Vanilla Bayesian Optimization Performs Great in High Dimensions. In Proceedings Of The 41st International Conference On Machine Learning; PMLR, 2024, pp. 20793–20817.

[43] Weeratunge H., Robe D., Menzel A. (2023). Bayesian coarsening:
Rapid tuning of polymer model parameters. Rheol.
Acta.

[44] Sestito J. M., Thatcher M. L., Shu L., Harris T. A. L., Wang Y. (2020). Coarse-Grained
Force Field Calibration Based on Multiobjective Bayesian Optimization
to Simulate Water Diffusion in Poly-*ϵ*-caprolactone. J. Phys. Chem. A.

[45] Prabhu J., Frigerio M., Petretto E., Campomanes P., Salentinig S., Vanni S. (2024). A Coarse-Grained SPICA Makeover for
Solvated and Bare Sodium and Chloride Ions. J. Chem. Theory Comput..

[46] Cordina R. J., Smith B., Tuttle T. (2023). COGITO: A Coarse-Grained
Force Field
for the Simulation of Macroscopic Properties of Triacylglycerides. J. Chem. Theory Comput..

[47] Eustache, R.-P. Handbook of Condensation Thermoplastic Elastomers; Fakirov, S. , Ed.; Wiley-VCH: Weinheim, Germany, 2005, pp. 263–280.

[48] Embaye A. S., Martínez-Izquierdo L., Malankowska M., Téllez C., Coronas J. (2021). Poly (ether-block-amide) copolymer
membranes in CO2 separation applications. Energy
Fuels.

[49] Khalilinejad I., Sanaeepur H., Kargari A. (2015). Preparation of poly (ether-6-block
amide)/PVC thin film composite membrane for CO2 separation: Effect
of top layer thickness and operating parameters. J. Membr. Sci. Res..

[50] Meshkat S., Kaliaguine S., Rodrigue D. (2018). Mixed matrix membranes based on amine
and non-amine MIL-53 (Al) in Pebax® MH-1657 for CO2 separation. Sep. Purif. Technol..

[51] Bernardo P., Clarizia G. (2020). Enhancing Gas Permeation Properties
of Pebax®
1657 Membranes via Polysorbate Nonionic Surfactants Doping. Polymers.

[52] Salestan S. K., Rahimpour A., Abedini R. (2021). Experimental and theoretical studies
of biopolymers on the efficient CO2/CH4 separation of thin-film Pebax®
1657 membrane. Chem. Eng. Process. Process Intensif..

[53] Liu Y.-C., Chen C.-Y., Lin G.-S., Chen C.-H., Wu K. C.-W., Lin C.-H., Tung K.-L. (2019). Characterization
and molecular simulation
of Pebax-1657-based mixed matrix membranes incorporating MoS2 nanosheets
for carbon dioxide capture enhancement. J. Membr.
Sci..

[54] Li X., Yu S., Li K., Ma C., Zhang J., Li H., Chang X., Zhu L., Xue Q. (2020). Enhanced gas separation
performance of Pebax mixed matrix membranes by incorporating ZIF-8
in situ inserted by multiwalled carbon nanotubes. Sep. Purif. Technol..

[55] Xin Q., Gao L., Ma F., Wang S., Xuan G., Ma X., Wei M., Zhang L., Zhang Y. (2023). Preparation of mixed matrix membrane
with high efficiency SO2 separation performance by photosensitive
modification and enhanced adsorption of metal–organic framework. J. Mater. Sci..

[56] Jiang X., Chuah C. Y., Goh K., Wang R. (2021). A facile direct
spray-coating
of Pebax® 1657: Towards large-scale thin-film composite membranes
for efficient CO2/N2 separation. J. Membr. Sci..

[57] Materials Design MedeA 3.5 (Materials Exploration and Design Analysis); 2022. www.materialsdesign.com.

[58] Sun H. (1998). COMPASS: An
ab Initio Force-Field Optimized for Condensed-Phase Applications Overview
with Details on Alkane and Benzene Compounds. J. Phys. Chem. B.

[59] Mejía A., Müller E. A., Chaparro Maldonado G. (2021). SGTPy: A Python code for calculating
the interfacial properties of fluids based on the square gradient
theory using the SAFT-VR Mie equation of state. J. Chem. Inf. Model..

[60] Ervik Å., Mejia A., Müller E. A. (2016). Bottled
SAFT: A web app providing
SAFT-*γ* Mie force field parameters for thousands
of molecular fluids. J. Chem. Inf. Model..

[61] Jiménez-Serratos G., Herdes C., Haslam A. J., Jackson G., Müller E. A. (2017). Group Contribution
Coarse-Grained Molecular Simulations of Polystyrene Melts and Polystyrene
Solutions in Alkanes Using the SAFT-*γ* Force
Field. Macromolecules.

[62] Richards, E. Coarse Grained Models of Surfactants; Ph.D. Thesis, Imperial College: London, 2022.

[63] Lafitte T., Avendaño C., Papaioannou V., Galindo A., Adjiman C. S., Jackson G., Müller E. A. (2012). SAFT-*γ* force
field for the simulation of molecular fluids: 3. Coarse-grained models
of benzene and hetero-group models of n-decylbenzene. Mol. Phys..

[64] Fayaz-Torshizi M., Müller E. A. (2021). Coarse-grained molecular dynamics
study of the self-assembly
of polyphilic bolaamphiphiles using the SAFT-*γ* Mie force field. Mol. Syst. Des. Eng..

[65] Patrone P. N., Dienstfrey A., Browning A. R., Tucker S., Christensen S. (2016). Uncertainty
quantification in molecular dynamics studies of the glass transition
temperature. Polymer.

[66] Watanabe, S. Tree-structured Parzen estimator: Understanding its algorithm components and their roles for better empirical performance arXiv 2023 10.48550/arXiv.2304.11127

[67] Garnett, R. Bayesian Optimization; Cambridge University Press, 2023.

[68] Akiba, T. ; Sano, S. ; Yanase, T. ; Ohta, T. ; Koyama, M. Optuna: A Next-generation Hyperparameter Optimization Framework. In Proceedings Of The 25th ACM SIGKDD International Conference On Knowledge Discovery And Data Mining; ACM, 2019, pp. 2623–2631.

[69] Priem R., Diouane Y., Bartoli N., Dubreuil S., Saves P. (2025). High-Dimensional
Bayesian Optimization Using Both Random and Supervised Embeddings. AIAA J..

[70] Rafiei, S. M. R. ; Amirahmadi, A. ; Griva, G. , Chaos rejection and optimal dynamic response for boost converter using SPEA multi-objective optimization approach. In 2009 35th Annual Conference of IEEE Industrial Electronics; IEEE, 2009, pp. 3315–3322.

[71] Pllana, S. ; Memeti, S. ; Kolodziej, J. , Customizing Pareto Simulated Annealing for Multi-Objective Optimization of Control Cabinet Layout. In 2019 22nd International Conference on Control Systems and Computer Science (CSCS); IEEE, 2019, pp. 78–85.

[72] Nguyen H. A., van Iperen Z., Raghunath S., Abramson D., Kipouros T., Somasekharan S. (2017). Multi-objective
optimization in scientific workflow. Procedia
Comput. Sci..

[73] Suter J. L., Müller W. A., Vassaux M., Anastasiou A., Simmons M., Tilbrook D., Coveney P. V. (2025). Rapid, Accurate
and Reproducible Prediction of the Glass Transition Temperature Using
Ensemble-Based Molecular Dynamics Simulation. J. Chem. Theory Comput..

