# Supplementary material for: Bayesian Optimization for High-Dimensional Coarse-Grained Model Parameterization: A Case Study on Pebax Polymer
Source: J Chem Theory Comput. 2026 Jan 21;22(5):2358–68. doi: 10.1021/acs.jctc.5c01500 (PMC12980707; doi:10.1021/acs.jctc.5c01500)
Supplement: Supplementary file 1 [file ct5c01500_si_001.pdf]

Supporting Information: Bayesian Optimization for High-Dimensional Coarse-Grained Model Parameterization: A Case Study on Pebax Polymer

Carlos A. Martins Junior,[†] Daniela A. Damasceno,[‡] Keat Yung Hue,[¶,§] Caetano R. Miranda,[*,†] Erich A. Müller,[¶] and Rodrigo A. Vargas-Hernández[‖,⊥]

†*University of São Paulo, Department of Materials Physics and Mechanics, Institute of Physics, Rua do Matão 1371, São Paulo, 05508-090, Brazil*

‡*University of São Paulo, Department of Mechatronics and Mechanical Systems Engineering, Polytechnic School, Av. Professor Mello Moraes, 2231, São Paulo, 05315-970, Brazil*

¶*Imperial College London, Department of Chemical Engineering, South Kensington Campus, London, SW7 2AZ, UK*

§*PETRONAS Research Sdn. Bhd., Lot 3288 & 3289, Off Jalan Ayer Itam, Kawasan Institusi Bangi, 43000 Kajang, Selangor, Malaysia*

‖*Department of Chemistry and Chemical Biology, McMaster University, 1280 Main Street West Hamilton, Ontario, L8S 4M1, Canada*

⊥*Brockhouse Institute for Materials Research, McMaster University, 1280 Main Street West Hamilton, Ontario, L8S 4M1, Canada*

E-mail: crmiranda@usp.br

The purpose of this Supplemental Material is to provide additional details about the proposed work in the main draft. The following sections contain all numerical details of the molecular dynamics (MD) simulations and optimization. We also report the values found for the optimal parameters in Section 2.

1 Numerical details of the Molecular Dynamics Simulations

The central goal of this work is to highlight the use of Bayesian optimization (BO) for the development of CG models. The optimization process, as described in the main text, depends on a total error function ($\mathcal{L}(\boldsymbol{\theta})$) that quantifies the discrepancy between the physical properties of the predicted target, given a set of parameters ($\boldsymbol{\theta}$), and the targets,

$$\mathcal{L}(\boldsymbol{\theta}) = w_\rho \mathcal{L}_\rho(\boldsymbol{\theta}) + w_{Rg} \mathcal{L}_{Rg}(\boldsymbol{\theta}) + w_{Tg} \mathcal{L}_{Tg}(\boldsymbol{\theta}), \quad (1)$$

where $\mathcal{L}_\rho$, $\mathcal{L}_{Rg}$ and $\mathcal{L}_{Tg}$ are the respective relative errors for the density (ρ), radius of gyration (Rg), and the glass transition temperature (Tg). The weights, $[w_\rho, w_{R_g}, w_{T_g}]$, in the objective function were chosen to balance the contributions of each term, considering the differences in the number of data points used for their calculations. Specifically, the term associated with Rg was multiplied by $1,875$, a factor derived from the ratio of $15,000$ (the number of points used for density calculation) to 8 (the number of points used for Rg). For the glass transition temperature, which depends on a single value, the weight w_{Tg} was set to $15,000$ due to the magnitude of $\mathcal{L}_{Tg}$.

Each evaluation of $\mathcal{L}$ at the suggested values of $\boldsymbol{\theta}$ by BO follows an iterative loop of molecular dynamics simulations, where the density, radius of gyration, and glass transition temperature are computed. Then the new values collected of $\mathcal{L}(\boldsymbol{\theta}_i)$ and $\boldsymbol{\theta}_i$ are used to update the Tree-Structured Parzen Estimator (TPE) model,[S1] which will be used in the

next iteration of BO to propose a candidate $\boldsymbol{\theta}$ which could lead to a lower value in $\mathcal{L}$. All parameters of the CG model were jointly optimized, that is $\boldsymbol{\theta}_i$ includes the bond length (l), bond constant (K_l), bond angles (θ), angle constants (k_θ), and Mie potential parameters (ϵ, σ, and γ_r). The TPE model and BO were implemented using the Optuna package,[S2] with a custom Python program that allows the use of the LAMMPS package for all required MD simulations. To ensure the robustness of the proposed scheme, we performed three independent BO runs, and the optimal parameters found by BO are reported in Tables S1-S7.

In the following, we further describe the numerical details in the simulations of the density, glass transition temperature, and radius of gyration. For density calculation, ρ, a simulation box measuring 55 × 55 × 90 Å containing 450 Pebax chains was used. The protocol began with an equilibration phase starting at 500 K, performed using a Langevin integrator, followed by cooling to 300 K and then to 150 K in the NPT ensemble with the Nosé-Hoover integrator. After equilibration, a production phase was conducted, also in the NPT ensemble.

At each temperature, $T_i = \{150, 175, 200, 225\}$ and $T_i = \{425, 450, 475, 500\}$ K, density values were measured after 0.3 ns of simulation. The system was gradually heated to reach the next target temperature. A time step of 15 femtoseconds was used. Both time step and simulation durations were determined by monitoring the convergence of density values. The resulting density values were used to fit two linear equations (Fig. S1), producing 7,500 target points for each fit. These points were then used to compute the error between the trial and the atomistic model. We also used these fits to obtain the glass temperature,

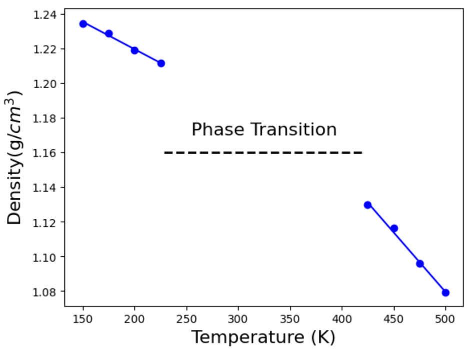

Figure S1: Density values (dots) and the corresponding linear fits used to determine the target property related to density. Data points near the glass transition temperature were excluded due to their non-linear behavior, which would compromise the accuracy of a linear approximation

following the procedure described in Ref.[S3] In this
procedure, Tg is obtained from the intersection of the two linear fits.

We obtained the radius of gyration by simulating only a single chain of Pebax in an almost $30 \times 30 \times 30$ Å simulation box. The equilibration phase was kept the same, as the procedure used for ρ. However, here we evaluated Rg for a wider range of temperatures, starting from 150 K to 500 K, incrementing every 25 K. Given the sensitivity of MD simulations of the Rg for a single Pebax, the duration of the chosen timestep, the MD simulations at each temperature were 0.2 ns long with a time step of 0.1 fs.

The atomistic simulations followed, in overall, the same procedure. The molecular dynamics simulations were performed using the LAMMPS package with the real unit system, periodic boundary conditions in all directions, and a fully atomistic representation. Interatomic interactions were described using the OPLS force field, as defined in the parameter file, which is hosted in `https://github.com/camjjr/bo_cgff/Atomistic_PEBAX_50chains`. Non-bonded interactions were modeled using a Lennard-Jones potential combined with long-range electrostatics treated by the PPPM method with a cutoff of 9.5 Å. The system, consisting of 3450 atoms within a cubic simulation box of 32.7 Å per side, was initialized from a pre-equilibrated structure file containing 16 atom types and 21 bond types. The equations of motion were integrated using a time step of 0.1 fs, with neighbor lists updated every step. Temperature and pressure control were applied through Nosé–Hoover thermostat and barostat schemes, with damping constants of 10 fs and 100 fs, respectively.

2 CG-BO Optimal Parameters

Tables S1-S7 show the optimal parameters from both the hybrid strategy and Bayesian Optimization.

Table S1: Optimal parameters for K_θ

K_θ	Hybrid Strategy (kcal/(mol rad^2))	BO (kcal/(mol rad^2))
T1-T2-T3	2.725	5.297
T1-T2-T3	6.582	7.333
T1-T3-T2	4.832	11.997
T2-T1-T3	5.440	6.772
T2-T3-T4	3.108	11.235
T4-T5-T5	3.408	6.555
T5-T5-t5	2.556	1.708

Table S2: Optimal parameters for θ

θ	Hybrid Strategy (rad)	BO (rad)
T1-T2-T3	2.393	2.134
T1-T2-T3	1.782	1.715
T1-T3-T2	2.347	1.512
T2-T1-T3	1.890	2.898
T2-T3-T4	2.353	1.279
T4-T5-T5	2.383	2.276
T5-T5-t5	2.389	3.012

Table S3: Optimal parameters for K_l

K_l	Hybrid Strategy (kcal/(molÅ))	BO (kcal/(molÅ))
T1-T2	6.178	4.874
T1-T3	4.922	4.129
T2-T3	10.86	9.186
T3-T4	7.120	6.554
T4-T5	5.394	5.822
T5-T5	9.352	1.884

Table S4: Optimal parameters for bond length. For the hybrid strategy, the bond length $\bar{l}_{ij}$ between two sequential beads i and j is set as $\bar{l}_{ij} = (\sigma_i + \sigma_j)/2$.

Bond Length ($\bar{l}$)	Hybrid Strategy (Å)	BO (Å)
T1-T2	3.70	4.04
T1-T3	4.08	4.797
T2-T3	3.60	3.738
T3-T4	3.71	4.004
T4-T5	3.65	2.506
T5-T5	3.86	4.476

Table S5: Optimal parameters for ϵ

ϵ	Hybrid Strategy (kcal/mol)	BO (kcal/mol)
T1	0.673	1.453
T2	0.749	1.440
T3	1.262	1.391
T4	0.507	1.169
T5	0.606	0.512

Table S6: Optimal parameters for σ

σ	Hybrid Strategy (Å)	BO (Å)
T1	3.220	3.940
T2	4.180	4.553
T3	3.989	2.847
T4	3.440	4.094
T5	3.860	4.773

Table S7: Optimal parameters for γ_r

γ	Hybrid Strategy	BO
T1	17.850	15.837
T2	16.400	15.629
T3	34.600	10.170
T4	12.890	11.503
T5	12.580	11.939

3 Effect of w_ρ, w_{Rg} and w_{Tg} in $\mathcal{L}(\boldsymbol{\theta})$

To better understand the optimization behavior and its dependence on the weighting scheme, we analyzed the convergence of three independent BO runs using different sets of weights: the original configuration $\mathbf{w} = [w_\rho, w_{Rg}, w_{Tg}]$ with $\mathbf{w} = [1,\ 1875,\ 1.5 \times 10^4]$, and two modified versions, $\mathbf{w}_2 = [10,\ 1875,\ 1.5 \times 10^3]$ and $\mathbf{w}_3 = [5,\ 18750,\ 1.5 \times 10^3]$. Fig. S2 shows the evolution of the logarithm of the total objective function for the $\mathbf{w}_2$ and $\mathbf{w}_3$ runs. In all three cases, the optimization converged to low-objective regions in fewer than 600 iterations. The inset histograms reveal that although most sampled points span a broad range of objective values, BO consistently targets a narrow region with significantly lower values. This behavior confirms BO's capacity to efficiently explore and exploit promising areas of the parameter space, even under varying weight configurations.

Beyond convergence, we assessed the physical accuracy of the optimized models by comparing the predicted glass transition temperature (Tg) with the atomistic reference. As shown in Table S8, all three BO-based models achieved substantially lower relative errors than the CG-Hybrid Strategy. In particular, even when the weights were altered, emphasizing density or downweighting Tg, the models optimized for BO maintained a relatively low error in predicting Tg. This could be attributed to intrinsic relationships among the physical properties, particularly between density and Tg, where the latter is derived from the slope change in the density curve. These findings demonstrate BO's robustness in identifying

parameter sets that generalize well across different objective function formulations.

Taken together, these results suggest that the structure of the search space, shaped by physical constraints, enables BO to consistently discover high-quality solutions. The limited sensitivity of the optimization outcomes to the specific weight configuration indicates that precise weight tuning is not a critical factor for BO's success. This reinforces the potential of Bayesian Optimization as a practical and scalable strategy for high-dimensional coarse-grained (CG) model development, with broader applicability to other complex material and molecular design tasks.

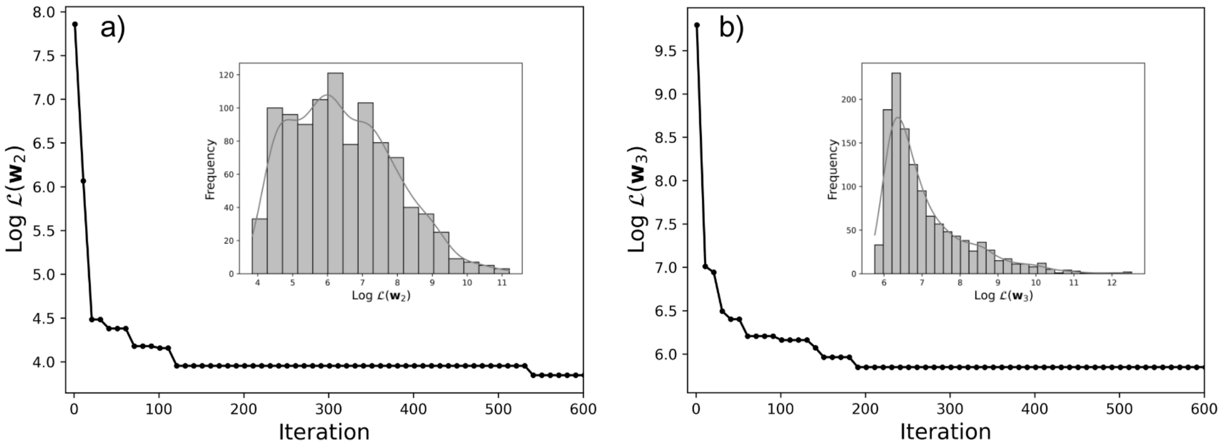

Figure S2: Convergence of Bayesian Optimization as a function of the number of iterations. Panel (A) shows the convergence for weight set $\mathbf{w}_2$, while panel (B) corresponds to weight set $\mathbf{w}_3$. In both plots, the black line represents the logarithm of the best objective value, $\log \mathcal{L}(\mathbf{w}_i)$ $(i = 2, 3)$, found up to each iteration. The inset in each figure shows a histogram of the sampled values of $\log \mathcal{L}(\mathbf{w}_i)$ $(i = 2, 3)$ from the BO run.

Table S8: Predicted glass transition temperature for the CG-BO model using three different weight sets $\mathbf{w}$, $\mathbf{w}_2$, and $\mathbf{w}_3$ and the CG-Hybrid Strategy. The atomistic model is used as the reference. This comparison highlights the robustness of Bayesian Optimization under different objective weightings.

Model	Tg (K)
Atomistic	378.10
CG - BO ($\mathbf{w}$)	336.69
CG - BO ($\mathbf{w_2}$)	295.16
CG - BO ($\mathbf{w_3}$)	341.09
CG - Hybrid Strategy	247.18

4 Principal Component Analysis of the Optimal Parameters

The Principal Component Analysis (PCA)[S4] was performed after the optimization process on the parameter trials. To ensure a balanced contribution of all parameters, they were normalized before the analysis, preventing any single parameter from dominating the variance. Additionally, bond angles were expressed in radians for consistency. The PCA implementation was carried out using the Scikit-learn package.[S5]

5 t-SNE

The t-distributed Stochastic Neighbor Embedding (t-SNE) analysis[S6] was performed after the optimization process to visualize the structure of the sampled parameters. The t-SNE projection was carried out using the Scikit-learn package.[S5]

References

(S1) Watanabe, S. Tree-structured Parzen estimator: Understanding its algorithm components and their roles for better empirical performance. arXiv preprint arXiv:2304.11127 **2023**,

(S2) Akiba, T.; Sano, S.; Yanase, T.; Ohta, T.; Koyama, M. Optuna: A Next-generation Hyperparameter Optimization Framework. Proceedings of the 25th ACM SIGKDD International Conference on Knowledge Discovery and Data Mining. 2019.

(S3) Patrone, P. N.; Dienstfrey, A.; Browning, A. R.; Tucker, S.; Christensen, S. Uncertainty quantification in molecular dynamics studies of the glass transition temperature. Polymer **2016**, 87, 246–259.

(S4) Halko, N.; Martinsson, P. G.; Tropp, J. A. Finding Structure with Randomness: Probabilistic Algorithms for Constructing Approximate Matrix Decompositions. SIAM Review **2011**, 53, 217–288.

(S5) Pedregosa, F.; Varoquaux, G.; Gramfort, A.; Michel, V.; Thirion, B.; Grisel, O.; Blondel, M.; Prettenhofer, P.; Weiss, R.; Dubourg, V.; Vanderplas, J.; Passos, A.; Cournapeau, D.; Brucher, M.; Perrot, M.; Duchesnay, E. Scikit-learn: Machine Learning in Python. Journal of Machine Learning Research **2011**, 12, 2825–2830.

(S6) Van der Maaten, L.; Hinton, G. Visualizing data using t-SNE. Journal of machine learning research **2008**, 9.
